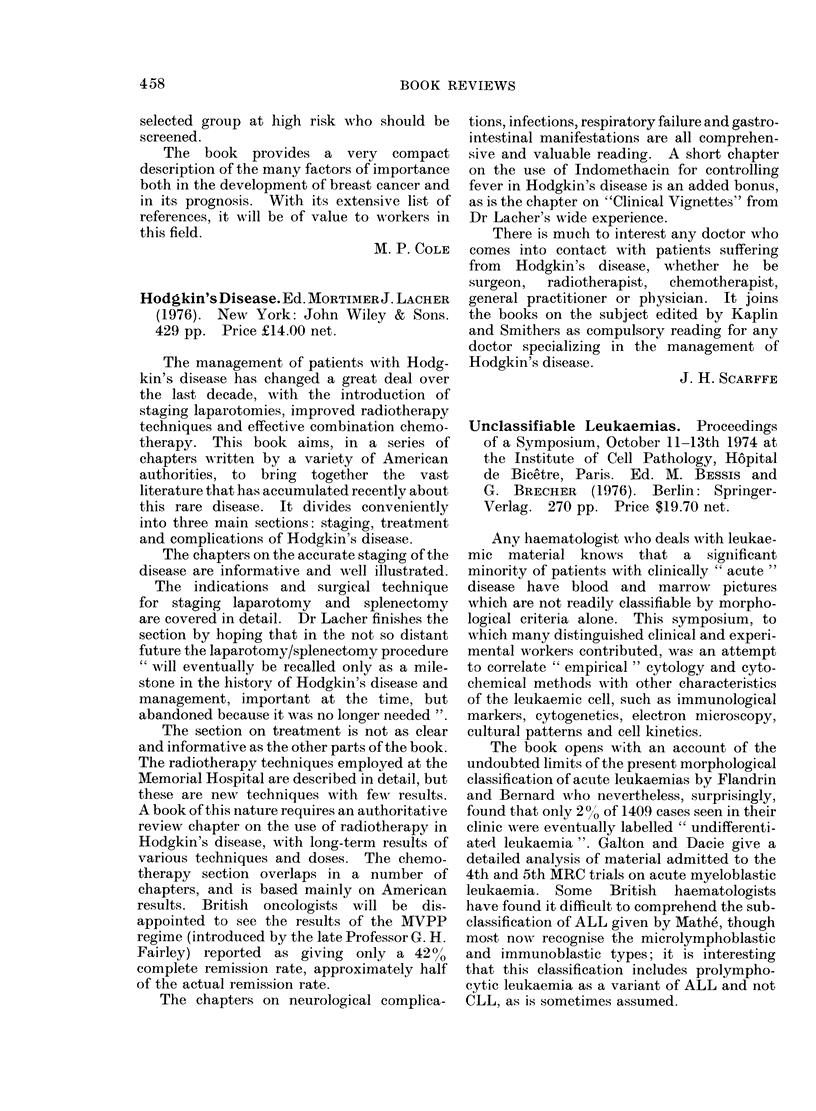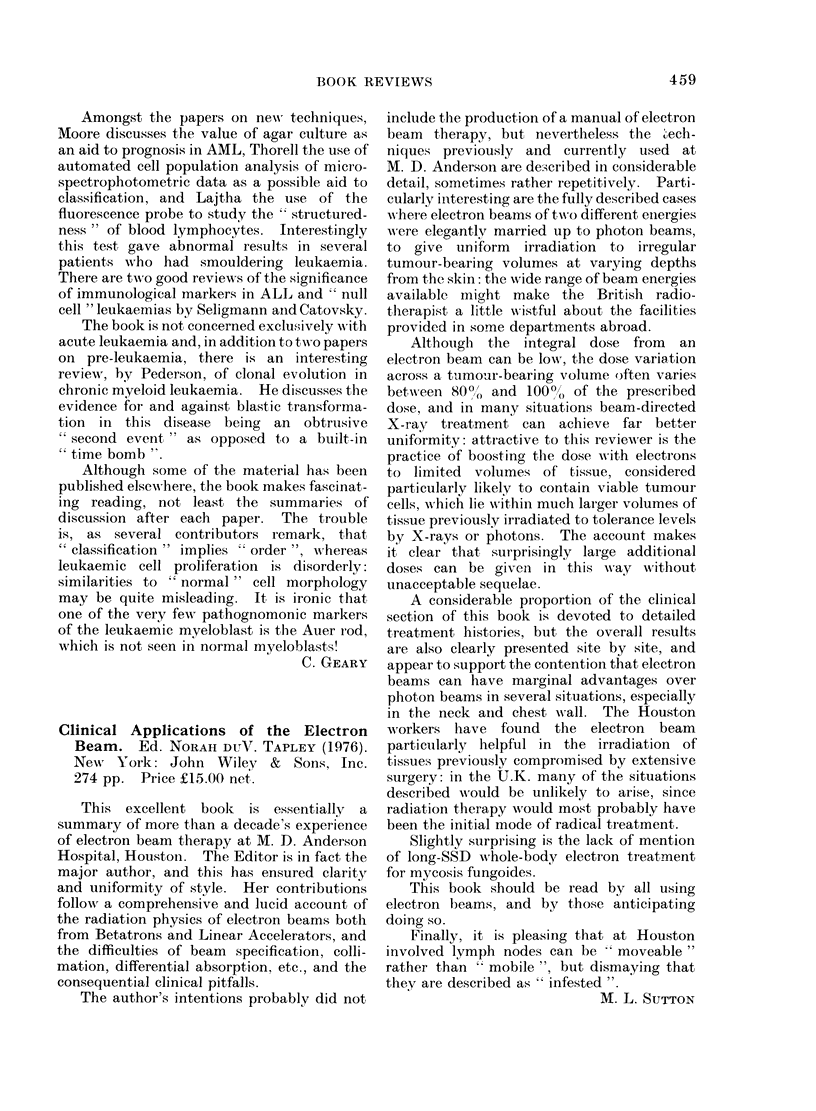# Unclassifiable Leukaemias

**Published:** 1976-10

**Authors:** C. Geary


**Unclassifiable Leukaemias.** Proceedings of a Symposium, October 11–13th 1974 at the Institute of Cell Pathology, Hôpital de Bicêtre, Paris. Ed. M. BESSIS and G. BRECHER (1976). Berlin: Springer-Verlag. 270 pp. Price $19.70 net.

Any haematologist who deals with leukaemic material knows that a significant minority of patients with clinically " acute " disease have blood and marrow pictures which are not readily classifiable by morphological criteria alone. This symposium, to which many distinguished clinical and experimental workers contributed, was an attempt to correlate " empirical " cytology and cytochemical methods with other characteristics of the leukaemic cell, such as immunological markers, cytogenetics, electron microscopy, cultural patterns and cell kinetics.

The book opens with an account of the undoubted limits of the present morphological classification of acute leukaemias by Flandrin and Bernard who nevertheless, surprisingly, found that only 2% of 1409 cases seen in their clinic were eventually labelled " undifferentiated leukaemia ". Galton and Dacie give a detailed analysis of material admitted to the 4th and 5th MRC trials on acute myeloblastic leukaemia. Some British haematologists have found it difficult to comprehend the subclassification of ALL given by Mathé, though most now recognise the microlymphoblastic and immunoblastic types; it is interesting that this classification includes prolymphocytic leukaemia as a variant of ALL and not CLL, as is sometimes assumed.

Amongst the papers on new techniques, Moore discusses the value of agar culture as an aid to prognosis in AML, Thorell the use of automated cell population analysis of microspectrophotometric data as a possible aid to classification, and Lajtha the use of the fluorescence probe to study the " structuredness " of blood lymphocytes. Interestingly this test gave abnormal results in several patients who had smouldering leukaemia. There are two good reviews of the significance of immunological markers in ALL and " null cell " leukaemias by Seligmann and Catovsky.

The book is not concerned exclusively with acute leukaemia and, in addition to two papers on pre-leukaemia, there is an interesting review, by Pederson, of clonal evolution in chronic myeloid leukaemia. He discusses the evidence for and against blastic transformation in this disease being an obtrusive " second event " as opposed to a built-in " time bomb ".

Although some of the material has been published elsewhere, the book makes fascinating reading, not least the summaries of discussion after each paper. The trouble is, as several contributors remark, that " classification " implies " order ", whereas leukaemic cell proliferation is disorderly: similarities to " normal " cell morphology may be quite misleading. It is ironic that one of the very few pathognomonic markers of the leukaemic myeloblast is the Auer rod, which is not seen in normal myeloblasts!

C. GEARY